# Closing Editorial to the Special Issue “Exploration and Innovation in Sustainable Rubber Performance”

**DOI:** 10.3390/polym18050576

**Published:** 2026-02-27

**Authors:** Pilar Bernal-Ortega, Anke Blume, Rafal Anyszka

**Affiliations:** Elastomer Technology & Engineering, Department of Mechanics of Solids, Surfaces & Systems (MS3), Faculty of Engineering Technology, University of Twente, Drienerlolaan 5, 7522 NB Enschede, The Netherlands; a.blume@utwente.nl (A.B.); r.p.anyszka@utwente.nl (R.A.)

It is with great pleasure that we present the collection of articles published in this Special Issue of MDPI’s *Polymers*, dedicated to “Exploration and Innovation in Sustainable Rubber Performance”. When we began this Special Issue, our goal was to gather innovative research addressing the ways in which rubber materials can be transformed to be more sustainable, whether that be through bio-based raw ingredients, improved recyclability, enhanced performance for extended durability, or novel strategies that reduce environmental footprints.

We are thrilled by the outcome: this issue features a diverse set of studies covering fundamental research, applied materials science, and engineering solutions. The contributions include investigations into recycled or bio-based fillers, novel processing methods, innovative elastomer formulations, studies on aging and durability, analyses of environmental impact, and explorations of material performance under real-world conditions. Together, they provide a complete overview of the current state of the art of the sustainability of rubber, reflecting both academic and industrial interests.

Through these works, this Special Issue addresses several key topics regarding sustainability within the rubber field and achieves the following results:

- Demonstrates viable pathways for integrating bio-based or recycled materials into elastomer matrices without compromising essential properties.
- Improves our understanding of structure–property relationships in sustainable rubber compounds, enabling the effective design of durable, high-performance materials.
- Highlights recycling, waste reduction, and life-cycle considerations for rubber products, contributing to circular economy approaches.
- Encourages innovation in filler, compounding, and processing techniques and collaborations across academia and industry.

We would like to extend our sincere gratitude to all authors, reviewers, and colleagues involved in the peer review and editorial process. Their dedication and scientific rigor have made this Special Issue possible. We also thank the Editorial Office of *Polymers* for their support and guidance throughout this process.

Finally, we hope that this collection will serve as a useful resource for researchers and industries aiming to push the boundaries of sustainable elastomer development. We encourage readers to explore the full set of papers and to build upon their findings, whether towards improved recycling processes, greener materials, or novel applications. We look forward to seeing how the insights gathered here will inspire future work and contribute to a more sustainable rubber industry.

